# Thermo-Electro-Mechanical Analysis of a Curved Functionally Graded Piezoelectric Actuator with Sandwich Structure

**DOI:** 10.3390/ma4122151

**Published:** 2011-12-12

**Authors:** Zhi Yan, Mostafa Zaman, Liying Jiang

**Affiliations:** Department of Mechanical and Materials Engineering, The University of Western Ontario, London, Ontario N6A 5B9, Canada; E-Mails: zyan25@uwo.ca (Z.Y.); mostafa_mecha@yahoo.com (M.Z.)

**Keywords:** functionally graded piezoelectric materials (FGPMs), curved actuator, sandwich structure, thermal effect, bimorph

## Abstract

In this work, the problem of a curved functionally graded piezoelectric (FGP) actuator with sandwich structure under electrical and thermal loads is investigated. The middle layer in the sandwich structure is functionally graded with the piezoelectric coefficient *g*_31_ varying continuously along the radial direction of the curved actuator. Based on the theory of linear piezoelectricity, analytical solutions are obtained by using Airy stress function to examine the effects of material gradient and heat conduction on the performance of the curved actuator. It is found that the material gradient and thermal load have significant influence on the electroelastic fields and the mechanical response of the curved FGP actuator. Without the sacrifice of actuation deflection, smaller internal stresses are generated by using the sandwich actuator with functionally graded piezoelectric layer instead of the conventional bimorph actuator. This work is very helpful for the design and application of curved piezoelectric actuators under thermal environment.

## 1. Introduction

Due to their excellent electromechanical coupling, fast response and design flexibility, piezoelectric ceramics have been regarded as promising materials for constructing various devices in micromechanical systems (MEMS), such as ultrasonic micromotors [1], actuators [2], micropumps and microvalves [3,4] and accelerometers [5], *etc*. Among these, piezoelectric bimorph and multimorph are commonly employed as fundamental elements to complement the functions of different devices. Usually, these structures are made of two or more layers of piezoelectric sheets and are jointed by bonding agents. However, such laminated piezoelectric structures suffer from high stress concentration near the interface due to the abrupt changes in both material composition and thermo-electro-elastic properties, which can cause severe deterioration of the bonding layer strength and reduce the lifetime of the structures. To overcome the drawbacks of laminated piezoelectric structures and meet some particular requirements for performance and reliability, the concept of functionally graded materials (FGMs) has been introduced into piezoelectric materials. This new class of materials is called functionally graded piezoelectric materials (FGPMs). As a result, a new type of sandwiched piezoelectric structure was developed with the middle layer functionally graded, *i.e.*, the middle layer has varying composition and properties, which is continuously jointed with the outer layers [6]. Therefore, the entire structure acts like a monomorph without any bonding agent and the failure caused by the interfacial debonding or stress concentration presented in the traditional laminated piezoelectric structures could be avoided. 

The fabrication and property investigation of FGPMs have attracted great attention from the research community. Among the early investigators, Zhu and Meng [6] developed FGM actuators based on PNN-PZT piezoelectric ceramics by the powder mould stacking press method. Wu *et al.* [7] fabricated a ceramic bimorph actuator with a smooth gradient by doping PZT with Zinc borate and demonstrated that the stresses induced are relatively uniform and do not peak in the center as conventional bimorphs. A laminated piezoelectric bimorph actuator with a graded compositional distribution of PZT and Pt was fabricated by Takagi *et al.* [8] using powder stacking and sintering. It was found that larger deflection and smaller stress were developed in this structure as compared to the conventional bimorph. For FGPMs in practical applications, the material gradient could exist in more than one material coefficient and the distribution of their material properties could be arbitrary. However, in order to make the analysis of FGPMs mathematically tractable, simplified models have been used to investigate the electromechanical behavior of FGP devices. The bending behavior of FGM actuators was predicted by Hauke *et al.* [9]. In their study, they used a simple analytical model in which the actuator is assumed to consist of *N* layers with stepwise linear piezoelectric coefficient in different layers, but elastic and dielectric coefficients are assumed to be constant. Based on the Kirchhoff-love hypothesis, Kruusing [10] presented some solutions of an FGP cantilever actuator and he also gave a brief review of design and modeling of these kinds of actuators. Using classical laminate theory, the electroelastic behavior of a piezoelectric composite actuator with functionally graded microstructure were analyzed in [11,12]. Huang *et al.* [13,14] derived the analytical solutions for FGP beams under both mechanical and electrical loads from the two-dimensional equations of piezoelectricity, in which the elastic, piezoelectric and dielectric coefficients of the piezoelectric beams were assumed to vary along the beam thickness direction only. By using stress function approach, Shi and his co-workers [15,16,17,18] obtained a set of exact solutions for the FGP cantilevers with varying piezoelectric parameter *g*_31_ or the elastic parameter *s*_33_ under different loading conditions.

Since some piezoelectric devices may operate under extreme environment with high temperature, the thermal effect could be a significant issue in the performance prediction and design of these devices. Some researchers have conducted studies on the electromechanical coupling of FGPMs with the consideration of thermal effect. Wang and Noda [19] developed a finite element code to study the functionally graded thermopiezoelectric composite structure and investigate how the functionally graded layer will affect the behavior of the composite structure. Based on Euler-Bernoulli theory, Joshi *et al.* [20] obtained exact solutions for the response of a laminated beam under thermal and electrical excitations, in which the structure consists of a substrate, an FGPM layer and an active piezoelectric layer. Lee [21] used a layerwise laminate theory and finite element formulation to examine the effect of material gradient on the response of thermo-electro-mechanical coupled piezoelectric bimorph actuators. Chen and Shi [22] derived exact solutions for an FGP cantilever with piezoelectric parameter *g*_31_ varying linearly along the thickness direction under different electrical and heat conduction conditions. Based on the theory of piezoelectricity, an FGP sandwich cantilever under electrical and thermal loads were studied in [23], in which all material parameters are assumed to vary in the direction of the thickness according to a power law distribution. Yang and Xiang [24] used the Timoshenko beam theory to investigate the static bending and dynamic response of FGP actuators under combined thermal-electro-mechanical loading.

It should be pointed out that most existing studies as mentioned above are on the flat FGP devices, which serve well on flat engineering structures. However, the applications of such flat devices on curved structures require the complicated shape of bonding layer and may significantly disturb the interfacial stress distribution and reduce the device precision. Thus curved FGP devices are more acceptable for applications in complex shaped structures, such as aircraft wings and satellite dishes [25]. Recently, researchers have attempted to study the electromechanical coupling behavior of curved FGP devices. Exact solutions for curved multi-layered piezoelectric and FGP actuators were obtained by Shi and his coworkers [26,27,28] with the assumption that only piezoelectric coefficient *g*_31_ varies along the radial direction of the circularly curved beam. However, there is very limited work of studying the thermal load effect in the curved FGP actuator configuration. The bending behavior of a circularly curved FGP cantilever actuator under an applied electrical load and heat conduction was investigated in our previous work [29]. It was found that thermal effect was significant on the electroelastic field of the curved actuator. To the authors’ best knowledge, there is no investigation of the thermal effect on the performance of sandwiched FGP structures thus far. It is, therefore, the objective of the current study to investigate the thermal-electro-elastic fields of a curved FGP actuator with sandwich structure under electrical and thermal loads. By using Airy stress function, analytical solutions are derived and numerical results are presented to show the effects of material gradients and thermal loads on the stresses, displacements, electric displacements and electric potential of the curved actuator. These results can also demonstrate the advantages of using the sandwiched FGP actuator over the traditional piezoelectric bimorph actuator. 

## 2. Formulation of the Problem

**Figure 1:**
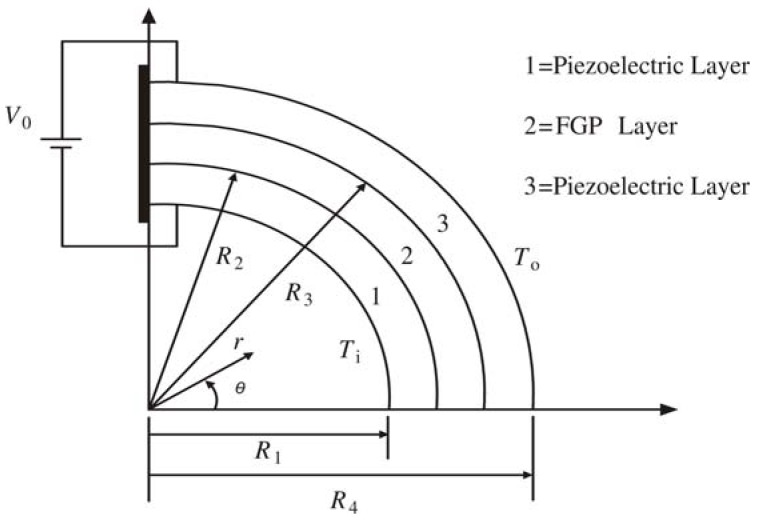
Schematic of curved FGP actuator with sandwiched structure.

The curved sandwich structure envisaged in the current work is fixed at one end and consists of three layers, with the lower and upper layers (layer 1 and layer 3) being two dissimilar homogeneous piezoelectric media and the middle layer being an FGP one (layer 2) as shown in Figure 1. The piezoelectric parameter *g*_31_ in the middle FGP layer is assumed to vary along the radial direction while approaches to the corresponding values of the homogeneous piezoelectric layers at the upper and lower surfaces, respectively. It is assumed that all the piezoelectric layers are poled in radial direction. For the analysis of this device, a polar coordinate system (*r*, *θ*) is used, and the thickness of the *k* th layer is determined by $h_{k}=R_{k+1}-R_{k}$ (*k* = 1~3), where *k* = 1, 2 and 3 refers to the lower layer, the FGP layer and the upper layer of the actuator. The actuator is subjected to an electric potential *V*_0_ between the outer surface of layer 3 and the inner surface of layer 1, and a thermal conduction occurs along the radial direction due to the temperature rise difference, *i.e.*, *T*_o_ on the outer surface of layer 3 and *T*_i_ on the inner surface of layer 1. 

In the absence of body forces and free charges, the equilibrium equations of the piezoelectric body are given by:

(1a) $$\frac{\partial \sigma_{rr}^{\left(k\right)}}{\partial r}+\frac{1}{r}\frac{\partial \sigma_{r\theta }^{\left(k\right)}}{\partial \theta }+\frac{\sigma_{rr}^{\left(k\right)}-\sigma_{\theta \theta }^{\left(k\right)}}{r}=0$$

(1b) $$\frac{\partial \sigma_{r\theta }^{\left(k\right)}}{\partial r}+\frac{1}{r}\frac{\partial \sigma_{\theta \theta }^{\left(k\right)}}{\partial \theta }+\frac{2\sigma_{r\theta }^{\left(k\right)}}{r}=0$$

(1c) $$\frac{1}{r}\frac{\partial D_{\theta }^{\left(k\right)}}{\partial \theta }+\frac{1}{r}D_{r}^{\left(k\right)}+\frac{\partial D_{r}^{\left(k\right)}}{\partial r}=0$$

where $\sigma_{ij}^{\left(k\right)}$ and $D_{i}^{\left(k\right)}$ (*i* = *r*, *θ*) are stress and electric displacement components, and the superscript “*k*” represents layer *k*. 

The constitutive equations of the piezoelectric media under plane strain condition can be written as:

(2a) $$\varepsilon_{\theta \theta }^{\left(k\right)}=s_{11}^{D(k)}\sigma_{\theta \theta }^{\left(k\right)}+s_{13}^{D(k)}\sigma_{rr}^{\left(k\right)}+g_{31}^{\left(k\right)}D_{r}^{\left(k\right)}+\alpha_{\theta }^{\left(k\right)}T^{\left(k\right)}$$

(2b) $$\varepsilon_{rr}^{\left(k\right)}=s_{13}^{D(k)}\sigma_{\theta \theta }^{\left(k\right)}+s_{33}^{D(k)}\sigma_{rr}^{\left(k\right)}+g_{33}^{\left(k\right)}D_{r}^{\left(k\right)}+\alpha_{r}^{\left(k\right)}T^{\left(k\right)}$$

(2c) $$\varepsilon_{r\theta }^{\left(k\right)}=s_{44}^{D(k)}\sigma_{r\theta }^{\left(k\right)}+g_{15}^{\left(k\right)}D_{\theta }^{\left(k\right)}$$

(2d) $$E_{\theta }^{\left(k\right)}=-g_{15}^{\left(k\right)}\sigma_{r\theta }^{\left(k\right)}+\zeta_{11}^{\left(k\right)}D_{\theta }^{\left(k\right)}$$

(2e) $$E_{r}^{\left(k\right)}=-g_{31}^{\left(k\right)}\sigma_{\theta \theta }^{\left(k\right)}-g_{33}^{\left(k\right)}\sigma_{rr}^{\left(k\right)}+\zeta_{33}^{\left(k\right)}D_{r}^{\left(k\right)}-\rho^{\left(k\right)}T^{\left(k\right)}$$

with $s_{ij}^{D(k)}$, $g_{ij}^{\left(k\right)}$, $\zeta_{ij}^{\left(k\right)}$, $\alpha_{i}^{\left(k\right)}$ and $\rho^{\left(k\right)}$ being the elastic, piezoelectric, dielectric, thermal expansion and pyroelectric coefficients, respectively. *T*(*k*) is the temperature rise. The strain $\varepsilon_{ij}^{\left(k\right)}$ and the electric field $E_{i}^{\left(k\right)}$ can be expressed in terms of the displacement components ($u_{r}^{\left(k\right)}$ and $u_{\theta }^{\left(k\right)}$) and the electric potential $\Phi^{\left(k\right)}$ as:

(3a) $$\varepsilon_{\theta \theta }^{\left(k\right)}=\frac{u_{r}^{\left(k\right)}}{r}+\frac{1}{r}\frac{\partial u_{\theta }^{\left(k\right)}}{\partial \theta }$$

(3b) $$\varepsilon_{rr}^{\left(k\right)}=\frac{\partial u_{r}^{\left(k\right)}}{\partial r}$$

(3c) $$\varepsilon_{r\theta }^{\left(k\right)}=\frac{1}{r}\frac{\partial u_{r}^{\left(k\right)}}{\partial \theta }+\frac{\partial u_{\theta }^{\left(k\right)}}{\partial r}-\frac{u_{\theta }^{\left(k\right)}}{r}$$

(3d) $$E_{\theta }^{\left(k\right)}=-\frac{1}{r}\frac{\partial \Phi^{\left(k\right)}}{\partial \theta }$$

(3e) $$E_{r}^{\left(k\right)}=-\frac{\partial \Phi^{\left(k\right)}}{\partial r}$$

To consider the thermal effects, the temperature is assumed to vary in the radial direction only. Then the steady state heat transfer equation can be reduced to a one-dimensional equation as:

(4) $$\frac{1}{r}\frac{\text{d}}{\text{d}r}\left(\kappa^{\left(k\right)}r\frac{\partial T^{\left(k\right)}}{\partial r}\right)=0$$

with $\kappa^{\left(k\right)}$ being the thermal conductivity coefficient.

To determine the temperature distribution in each layer of the actuator, the thermal boundary conditions at the lower and upper surfaces of the actuator are applied as:

(5) $$T^{\left(1\right)}(R_{1})=T_{\text{i}};\begin{array}{ccc} & & T^{\left(3\right)}(R_{4})=T_{\text{o}} \end{array}$$

and the continuity conditions of temperature and heat flow at the interfaces between adjacent layers are:

(6) $$T^{\left(1\right)}(R_{2})=T^{\left(2\right)}(R_{2});T^{\left(2\right)}(R_{3})=T^{\left(3\right)}(R_{3});r\kappa^{\left(1\right)}\frac{\text{d}T^{\left(1\right)}}{\text{d}r}{|}_{r=R_{2}}=r\kappa^{\left(2\right)}\frac{\text{d}T^{\left(2\right)}}{\text{d}r}{|}_{r=R_{2}};r\kappa^{\left(2\right)}\frac{\text{d}T^{\left(2\right)}}{\text{d}r}{|}_{r=R_{3}}=r\kappa^{\left(3\right)}\frac{\text{d}T^{\left(3\right)}}{\text{d}r}{|}_{r=R_{3}}$$

Then the temperature distribution in each layer of the curved piezoelectric structure can be obtained from Equations (4–6) as:

(7) $$T^{\left(k\right)}(r)=T_{\text{i}}+C\left(\sum_{i=1}^{k}\int_{R_{i}}^{R_{i+1}}\frac{1}{r\kappa^{\left(i\right)}}\text{d}r-\int_{R_{k}}^{R_{k+1}}\frac{1}{r\kappa^{\left(k\right)}}\text{d}r+\int_{R_{k}}^{r}\frac{1}{r\kappa^{\left(k\right)}}\text{d}r\right)\begin{array}{cc} & R_{k}<r<R_{k+1} \end{array}$$

where *C* is a constant described as:

(8) $$C=\frac{T_{\text{o}}-T_{\text{i}}}{\frac{1}{\kappa^{\left(1\right)}}\ln\frac{R_{2}}{R_{1}}+\frac{1}{\kappa^{\left(2\right)}}\ln\frac{R_{3}}{R_{2}}+\frac{1}{\kappa^{\left(3\right)}}\ln\frac{R_{4}}{R_{3}}}$$

It is obvious that the thermal conduction will affect the electroelastic field in the curved piezoelectric media, as shown in Equation (2).

For practical FGPMs, the distribution of material properties can be arbitrary, for example, the individual material coefficient may vary independently. However, it is difficult, if not impossible to get the analytical solution. Moreover, it is also found that the dependence on poling for elastic and dielectric coefficients is much less pronounced than that for the piezoelectric coefficient *g*_31_ [9,28,30]. Therefore, to make the analysis mathematically tractable, only the piezoelectric coefficient $g_{31}$ of the piezoelectric media is assumed to vary along the radial direction in the sandwich structure, while all the other material coefficients are assumed as constant. A Taylor series expansion is used to describe the arbitrary function *g*_31_(*r*) for the FGP layer in terms of the following *N* th-order polynomial function as [28]:

(9) $$g_{31}(r)=J_{0}+J_{1}r+J_{2}r^{2}+\cdots +J_{N}r^{N}=\sum_{i=0}^{N}J_{i}r^{i}\begin{array}{cc} & R_{2} \end{array}<r<R_{3}$$

where $J_{i}$(*i* = 0…*N*) are material constants. 

From Equation (9), it is seen that *g*_31_(*r*) could be of arbitrary format. For the case that the FGP is exponentially graded along the radial direction, *g*_31_ can be expressed as:

(10) $$g_{31}^{\left(2\right)}=g_{31}(r)=g_{0}e^{\beta r}\begin{array}{cc} & R_{2}<r<R_{3} \end{array}$$

where $g_{0}$ and β are material constant and the material gradient, which could be determined from:

(11) $$g_{0}=g_{31}^{\left(1\right)}{\left(\frac{g_{31}^{\left(1\right)}}{g_{31}^{\left(3\right)}}\right)}^{\frac{R_{2}}{R_{3}-R_{2}}}$$

and

(12) $$\beta =\frac{1}{R_{3}-R_{2}}\ln\left(\frac{g_{31}^{\left(3\right)}}{g_{31}^{\left(1\right)}}\right)$$

with $g_{31}^{\left(1\right)}$ and $g_{31}^{\left(3\right)}$ being the piezoelectric coefficients of the lower and upper piezoelectric layer, respectively. To get the analytical solutions for the curved FGP actuator, we expand the *g*_31_(*r*) using Taylor’s series expansion;

(13) $$g_{31}(r)=g_{0}\left(1+\beta r+\frac{\beta^{2}}{2!}r^{2}+\frac{\beta^{3}}{3!}r^{3}+\cdots +\frac{\beta^{N}}{N!}r^{N}+\cdots \right)$$

in which the coefficients $g_{0}\left(1,\beta ,\frac{\beta^{2}}{2!},\frac{\beta^{3}}{3!},\cdots \frac{\beta^{N}}{N!},\cdots \right)$ correspond to the parameters $\left(J_{0},J_{1},J_{2},J_{3},\cdots J_{N},\cdots \right)$ in Equation (9), respectively.

For the actuator as shown in Figure 1, it is obvious that the following boundary conditions are satisfied automatically:

(14) $$D_{\theta }{|}_{\theta =0=}D_{\theta }{|}_{\theta =\pi /2}=0$$

(15) $$\sigma_{r\theta }^{\left(1\right)}{|}_{r=R_{1}}=\sigma_{r\theta }^{\left(3\right)}{|}_{r=R_{4}}=0,\sigma_{r\theta }^{\left(1\right)}{|}_{r=R_{2}}=\sigma_{r\theta }^{\left(2\right)}{|}_{r=R_{2}},\sigma_{r\theta }^{\left(2\right)}{|}_{r=R_{3}}\sigma_{r\theta }^{\left(3\right)}{|}_{r=R_{3}}$$

Besides, the following mechanical and electrical boundary conditions as well as the continuity conditions at the interface of any two adjacent layers should also be satisfied:

I. Mechanical boundary conditions for stresses at the upper and lower surfaces

(16) $$\sigma_{rr}^{\left(1\right)}{|}_{r=R_{1}}\sigma_{rr}^{\left(3\right)}{|}_{r=R_{4}}=0$$

II. Electrical boundary conditions at the upper and lower surfaces

(17) $$\Phi^{\left(1\right)}{|}_{r=R_{1}}=0,\Phi^{\left(3\right)}{|}_{r=R_{4}}=V_{0}$$

III. Mechanical boundary conditions for displacements at the fixed end $\left(\theta =\frac{\pi }{2}\right)$

(18) $$u_{r}^{\left(2\right)}\left(R_{0},\frac{\pi }{2}\right)=0;u_{\theta }^{\left(2\right)}\left(R_{0},\frac{\pi }{2}\right)=0;\frac{\partial u_{r}^{\left(2\right)}\left(R_{0},\frac{\pi }{2}\right)}{\partial \theta }=0$$

where *R*_0_ is taken as the average radius of the actuator, *i.e.*, $R_{0}=\frac{R_{1}+R_{4}}{2}$.

IV. Continuity conditions for electric displacements

(19) $$D_{r}^{\left(1\right)}{|}_{r=R_{2}}=D_{r}^{\left(2\right)}{|}_{r=R_{2}},D_{r}^{\left(2\right)}{|}_{r=R_{3}}=D_{r}^{\left(3\right)}{|}_{r=R_{3}}$$

V. Continuity conditions for stresses

(20) $$\sigma_{rr}^{\left(1\right)}{|}_{r=R_{2}}=\sigma_{rr}^{\left(2\right)}{|}_{r=R_{2}},\sigma_{rr}^{\left(2\right)}{|}_{r=R_{3}}=\sigma_{rr}^{\left(3\right)}{|}_{r=R_{3}}$$

VI. Continuity conditions for displacements

(21) $$u_{r}^{\left(1\right)}{|}_{r=R_{2}}=u_{r}^{\left(2\right)}{|}_{r=R_{2}},u_{r}^{\left(2\right)}{|}_{r=R_{3}}=u_{r}^{\left(3\right)}{|}_{r=R_{3}}$$

(22) $$u_{\theta }^{\left(1\right)}{|}_{r=R_{2}}=u_{\theta }^{\left(2\right)}{|}_{r=R_{2}},u_{\theta }^{\left(2\right)}{|}_{r=R_{3}}=u_{\theta }^{\left(3\right)}{|}_{r=R_{3}}$$

VII. Continuity conditions for electric potential

(23) $$\Phi^{\left(1\right)}{|}_{r=R_{2}}=\Phi^{\left(2\right)}{|}_{r=R_{2}},\Phi^{\left(2\right)}{|}_{r=R_{3}}=\Phi^{\left(3\right)}{|}_{r=R_{3}}$$

Mechanical boundary conditions at the free end (θ=0)

(24) $$\int_{R_{1}}^{R_{4}}\sigma_{\theta \theta }\text{d}r=0,\int_{R_{1}}^{R_{4}}\sigma_{r\theta }\text{d}r=0,\int_{R_{1}}^{R_{4}}\sigma_{\theta \theta }r\text{d}r=0$$

## 3. Solution of the Problem

To find the solutions of Equations (1–3), an Airy stress function is introduced. For the considered plane problem of a curved beam with material coefficients only varying continuously along the radial direction, when only electric voltage is applied between the upper and lower surfaces of the beam with heat conduction through the thickness direction, both Airy stress function *Ψ* and electric potential *Φ* in each layer can be assumed as a function of *r*. Correspondingly, the stress components are expressed as:

(25) $$\sigma_{rr}^{\left(k\right)}=\frac{1}{r}\frac{\text{d}\psi^{\left(k\right)}}{\text{d}r};\sigma_{\theta \theta }^{\left(k\right)}=\frac{{\text{d}}^{\text{2}}\psi^{\left(k\right)}}{\text{d}r^{2}};\sigma_{r\theta }^{\left(k\right)}=0$$

and the electric field can be easily obtained from Equations (1–3) as:

(26) $$E_{\theta }^{\left(k\right)}=0;E_{r}^{\left(k\right)}=-\frac{\text{d}\Phi^{\left(k\right)}}{\text{d}r};D_{\theta }^{\left(k\right)}=0;D_{r}^{\left(k\right)}=\frac{C_{5}^{\left(k\right)}}{r}$$

in which $C_{5}^{\left(k\right)}$(*k*= 1~3) are constants to be determined. To ensure that the displacements can be obtained by integrating the strain fields, the following compatibility equations must be satisfied:

(27) $$\left(\frac{\partial^{2}}{\partial r^{2}}+\frac{2}{r}\frac{\partial }{\partial r}\right)\varepsilon_{\theta \theta }^{\left(k\right)}+\left(\frac{1}{r^{2}}\frac{\partial^{2}}{\partial \theta^{2}}-\frac{1}{r}\frac{\partial }{\partial r}\right)\varepsilon_{rr}^{\left(k\right)}=\left(\frac{1}{r^{2}}\frac{\partial }{\partial \theta }+\frac{1}{r}\frac{\partial^{2}}{\partial r\partial \theta }\right)\varepsilon_{r\theta }^{\left(k\right)}$$

Substituting Equations (2,7,9,25,26) into Equation (27), we have:

(28a) $$s_{11}^{D(k)}\frac{{\text{d}}^{\text{4}}\psi^{\left(k\right)}}{\text{d}r^{4}}+2\frac{s_{11}^{D(k)}{\text{d}}^{\text{3}}\psi^{\left(k\right)}}{r\text{d}r^{3}}-\frac{s_{33}^{D(k)}{\text{d}}^{\text{2}}\psi^{\left(k\right)}}{r^{2}\text{d}r^{2}}+\frac{s_{33}^{D(k)}\text{d}\psi^{\left(k\right)}}{r^{3}\text{d}r}+C_{5}^{\left(k\right)}\frac{g_{33}^{\left(k\right)}}{r^{3}}+\frac{C}{\kappa^{\left(k\right)}r^{2}}\left(\alpha_{\theta }^{\left(k\right)}-\alpha_{r}^{\left(k\right)}\right)=0(k=1,3)$$

and

(28b) $$s_{11}^{D(k)}\frac{{\text{d}}^{\text{4}}\psi^{\left(k\right)}}{\text{d}r^{4}}+2\frac{s_{11}^{D(k)}{\text{d}}^{\text{3}}\psi^{\left(k\right)}}{r\text{d}r^{3}}-\frac{s_{33}^{D(k)}{\text{d}}^{\text{2}}\psi^{\left(k\right)}}{r^{2}\text{d}r^{2}}+\frac{s_{33}^{D(k)}\text{d}\psi^{\left(k\right)}}{r^{3}\text{d}r}+C_{5}^{\left(k\right)}\sum_{i=2}^{N}J_{i}i(i-1)r^{i-3}+C_{5}^{\left(k\right)}\frac{g_{33}^{\left(k\right)}}{r^{3}}+\frac{C}{\kappa^{\left(k\right)}r^{2}}\left(\alpha_{\theta }^{\left(k\right)}-\alpha_{r}^{\left(k\right)}\right)=0\;,\;(k=2)$$

By solving Equations (28a) and (28b), we can obtain the Airy stress functions as follows:

(29a) $$\psi^{\left(k\right)}(r)=-G^{\left(k\right)}r+C_{1}^{\left(k\right)}+C_{2}^{\left(k\right)}r^{2}+C_{3}^{\left(k\right)}r^{-s^{\left(k\right)}+1}+C_{4}^{\left(k\right)}r^{s^{\left(k\right)}+1}+H^{\left(k\right)}r^{2}\ln r\;,\;(k=1,\;3)$$

and

(29b) $$\psi^{\left(k\right)}(r)=-G^{\left(k\right)}r+C_{1}^{\left(k\right)}+C_{2}^{\left(k\right)}r^{2}+C_{3}^{\left(k\right)}r^{-s^{\left(k\right)}+1}+C_{4}^{\left(k\right)}r^{s^{\left(k\right)}+1}+C_{5}^{\left(k\right)}f_{2}(r)+H^{\left(k\right)}r^{2}\ln r\;,\;(k=2)$$

where

(30) $$f_{2}(r)=\frac{1}{s_{11}^{D(2)}}\sum_{i=2}^{N}\frac{i}{i+1}\frac{J_{i}}{{\left(s^{\left(2\right)}\right)}^{2}-i^{2}}r^{i+1};\;G^{\left(k\right)}=\frac{C_{5}^{\left(k\right)}g_{33}^{\left(k\right)}}{s_{33}^{D(k)}};\;H^{\left(k\right)}=\frac{C\left(\alpha_{\theta }^{\left(k\right)}-\alpha_{r}^{\left(k\right)}\right)}{2\kappa^{\left(k\right)}s_{11}^{D(k)}\left({\left(s^{\left(k\right)}\right)}^{2}-1\right)};\;s^{\left(k\right)}=\sqrt{\frac{s_{33}^{D(k)}}{s_{11}^{D(k)}}}$$

and $C_{i}^{\left(k\right)}$(*k*=1~3 and *i*=1~5) are unknown constants to be determined from boundary conditions. However, $C_{1}^{\left(k\right)}$ do not need to be considered, since these coefficients in the Airy stress functions obviously have no influence on the electroelastic fields of the curved actuator. For the material properties considered in the current study, it is also seen from Equation (30) that $s^{\left(1\right)}=s^{\left(2\right)}=s^{\left(3\right)}=s$ and $H^{\left(1\right)}=H^{\left(2\right)}=H^{\left(3\right)}=H$.

Substituting Equations (29a) and (29b) into Equation (25), the stress components in each layer can be determined as:

(31a) $$\sigma_{rr}^{\left(k\right)}=-\frac{G^{\left(k\right)}}{r}+2C_{2}^{\left(k\right)}+(-s+1)r^{-s-1}C_{3}^{\left(k\right)}+(s+1)r^{s-1}C_{4}^{\left(k\right)}+H(2\ln r+1)$$

(31b) $$\sigma_{\theta \theta }^{\left(k\right)}=2C_{2}^{\left(k\right)}-s(-s+1)r^{-s-1}C_{3}^{\left(k\right)}+s(s+1)r^{s-1}C_{4}^{\left(k\right)}+H(3+2\ln r)\;,\;(k=1,\;3)$$

and

(32a) $$\sigma_{rr}^{\left(k\right)}=-\frac{G^{\left(k\right)}}{r}+2C_{2}^{\left(k\right)}+(-s+1)r^{-s-1}C_{3}^{\left(k\right)}+(s+1)r^{s-1}C_{4}^{\left(k\right)}+C_{5}^{\left(k\right)}\frac{f_{2}^{\prime }(r)}{r}+H(2\ln r+1)$$

(32b) $$\sigma_{\theta \theta }^{\left(k\right)}=2C_{2}^{\left(k\right)}-s(-s+1)r^{-s-1}C_{3}^{\left(k\right)}+s(s+1)r^{s-1}C_{4}^{\left(k\right)}+C_{5}^{\left(k\right)}f_{2}^{\prime \prime }(r)+H(3+2\ln r)\;,\;(k=2)$$

where $f_{2}^{\prime }(r)$ and $f_{2}^{\prime \prime }(r)$ are the first and second derivatives of $f_{2}(r)$. From Equations (2,3,31,32), the displacements and electric potential for the layer *k* can be determined as:

(33a) $$\begin{array}{cc} \begin{array}{l} u_{r}^{\left(k\right)}=s_{13}^{D(k)}\left[2rC_{2}^{\left(k\right)}+(-s+1)r^{-s}C_{3}^{\left(k\right)}+(s+1)r^{s}C_{4}^{\left(k\right)}+H(2r\ln r+r)\right]\\ +s_{33}^{D(k)}\left[2rC_{2}^{\left(k\right)}-\frac{-s+1}{s}r^{-s}C_{3}^{\left(k\right)}+\frac{s+1}{s}r^{s}C_{4}^{\left(k\right)}+H(2r\ln r-r)\right]\\ +\alpha_{r}^{\left(k\right)}r\left[T^{\left(k\right)}(r)-\frac{C}{\kappa^{\left(k\right)}}\right]+D_{1}^{\left(k\right)}\cos\theta +D_{2}^{\left(k\right)}\sin\theta -s_{13}^{D(k)}G^{\left(k\right)}+g_{31}^{\left(k\right)}C_{5}^{\left(k\right)} \end{array} & \left(k=1,\;3\right) \end{array}$$

(33b) $$\begin{array}{cc} \begin{array}{l} u_{\theta k}=s_{11}^{D(k)}\left[2rC_{2}^{\left(k\right)}+H(2r\ln r+3r)\right]\theta -s_{33}^{D(k)}\left[2rC_{2}^{\left(k\right)}+H(2r\ln r-r)\right]\theta \\ +\left(\alpha_{\theta }^{\left(k\right)}-\alpha_{r}^{\left(k\right)}\right)r\theta T^{\left(k\right)}(r)+\alpha_{r}^{\left(k\right)}r\theta \frac{C}{\kappa^{\left(k\right)}}-\sin\theta D_{1}^{\left(k\right)}+\cos\theta D_{2}^{\left(k\right)}+rD_{3}^{\left(k\right)} \end{array} & \left(k=1,\;3\right) \end{array}$$

(33c) $$\begin{array}{cc} \begin{array}{l} \Phi^{\left(k\right)}=g_{31}^{\left(k\right)}\left[2rC_{2}^{\left(k\right)}+(-s+1)r^{-s}C_{3}^{\left(k\right)}+(s+1)r^{s}C_{4}^{\left(k\right)}+H(2r\ln r+r)\right]\\ +g_{33}^{\left(k\right)}\left[-G^{\left(k\right)}\ln r+2rC_{2}^{\left(k\right)}-\frac{-s+1}{s}r^{-s}C_{3}^{\left(k\right)}+\frac{s+1}{s}r^{s}C_{4}^{\left(k\right)}+H(2r\ln r-r)\right]\\ -\zeta_{33}^{\left(k\right)}\ln rC_{5}^{\left(k\right)}+\rho^{\left(k\right)}r\left[T^{\left(k\right)}(r)-\frac{C}{\kappa^{\left(k\right)}}\right]+C_{6}^{\left(k\right)} \end{array} & (k=1,\;3 \end{array})$$

and

(34a) $$\begin{array}{cc} \begin{array}{l} u_{r}^{\left(k\right)}=s_{13}^{D(k)}\left[2rC_{2}^{\left(k\right)}+(-s+1)r^{-s}C_{3}^{\left(k\right)}+(s+1)r^{s}C_{4}^{\left(k\right)}+H(2r\ln r+r)+C_{5}^{\left(k\right)}f_{2}^{\prime }(r)\right]\\ +s_{33}^{D(k)}\left[2rC_{2}^{\left(k\right)}-\frac{-s+1}{s}r^{-s}C_{3}^{\left(k\right)}+\frac{s+1}{s}r^{s}C_{4}^{\left(k\right)}+H(2r\ln r-r)+C_{5}^{\left(k\right)}f_{3}(r)\right]\\ +\alpha_{r}^{\left(k\right)}r\left[T^{\left(k\right)}(r)-\frac{C}{\kappa^{\left(k\right)}}\right]+D_{1}^{\left(k\right)}\cos\theta +D_{2}^{\left(k\right)}\sin\theta -s_{13}^{D(k)}G^{\left(k\right)}+J_{0}C_{5}^{\left(k\right)} \end{array} & \left(k=2\right) \end{array}$$

(34b) $$\begin{array}{cc} \begin{array}{l} u_{\theta }^{\left(k\right)}=s_{11}^{D(k)}\left[2rC_{2}^{\left(k\right)}+H(2r\ln r+3r)\right]\theta -s_{33}^{D(k)}\left[2rC_{2}^{\left(k\right)}+H(2r\ln r-r)\right]\theta \\ +\left(\alpha_{\theta }^{\left(k\right)}-\alpha_{r}^{\left(k\right)}\right)r\theta T^{\left(k\right)}(r)+\alpha_{r}^{\left(k\right)}r\theta \frac{C}{\kappa^{\left(k\right)}}-\sin\theta D_{1}^{\left(k\right)}+\cos\theta D_{2}^{\left(k\right)}+rD_{3}^{\left(k\right)}+C_{5}^{\left(k\right)}J_{1}r\theta \end{array} & \left(k=2\right) \end{array}$$

(34c) $$\begin{array}{cc} \begin{array}{l} \Phi^{\left(k\right)}=\sum_{i=0}^{N}\left[2\frac{J_{i}}{i+1}r^{i+1}C_{2}^{\left(k\right)}-s(-s+1)\frac{J_{i}}{i-s}r^{i-s}C_{3}^{\left(k\right)}+s(s+1)\frac{J_{i}}{i+s}r^{i+s}C_{4}^{\left(k\right)}+\frac{HJ_{i}}{i+1}r^{i+1}\left(2\ln r+3-\frac{2}{i+1}\right)\right]\\ +g_{33}^{\left(k\right)}\left[-G^{\left(k\right)}\ln r+2rC_{2}^{\left(k\right)}-\frac{-s+1}{s}r^{-s}C_{3}^{\left(k\right)}+\frac{s+1}{s}r^{s}C_{4}^{\left(k\right)}+H(2r\ln r-r)+C_{5}^{\left(k\right)}f_{3}(r)\right]\\ -\zeta_{33}^{\left(k\right)}\ln rC_{5}^{\left(k\right)}+f_{4}(r)C_{5}^{\left(k\right)}+\rho^{\left(k\right)}r\left[T^{\left(k\right)}(r)-\frac{C}{\kappa^{\left(k\right)}}\right]+C_{6}^{\left(k\right)} \end{array} & \left(k=2\right) \end{array}$$

in which

(35) $$f_{3}(r)=\frac{1}{s_{11}^{D(2)}}\sum_{i=2}^{N}\frac{J_{i}}{s^{2}-i^{2}}r^{i};\;f_{4}(r)=\frac{1}{s_{11}^{D(2)}}\int \left(\sum_{i=2}^{N}\frac{J_{i}i^{2}}{s^{2}-i^{2}}r^{i-1}\sum_{i=0}^{N}J_{i}r^{i}\right)\text{d}r$$

and $C_{6}^{\left(k\right)}$ and $D_{j}^{\left(k\right)}$ (*j*, *k*=1~3) are unknown constants to be determined from boundary conditions given in Section 2. 

Substituting the electric displacements, stresses, electric potentials and displacement fields in Equations (31–34) into the boundary conditions (16–24) in the previous Section, the unknown constants can be obtained after lengthy derivations as follows:

(36) $$C_{5}^{\left(1\right)}=C_{5}^{\left(2\right)}=C_{5}^{\left(3\right)}=C_{5},D_{1}^{\left(1\right)}=D_{1}^{\left(2\right)}=D_{1}^{\left(3\right)}=D_{1},D_{2}^{\left(1\right)}=D_{2}^{\left(2\right)}=D_{2}^{\left(3\right)}=D_{2},D_{3}^{\left(1\right)}=D_{3}^{\left(2\right)}=D_{3}^{\left(3\right)}=D_{3}$$

(37) $$Y=P^{-1}X$$

with

$$P=\left\{\begin{array}{llllllllll} 2R_{1} & A_{1} & B_{1} & 0 & 0 & 0 & 0 & 0 & 0 & L_{1}\\ 0 & 0 & 0 & 0 & 0 & 0 & 2R_{4} & A_{3}^{\prime } & B_{3}^{\prime } & L_{2}\\ 2R_{2} & A_{1}^{\prime } & B_{1}^{\prime } & -2R_{2} & -A_{2} & -B_{2} & 0 & 0 & 0 & L_{3}\\ 0 & 0 & 0 & 2R_{3} & A_{2}^{\prime } & B_{2}^{\prime } & -2R_{3} & -A_{3} & -B_{3} & L_{4}\\ E_{1}R_{2} & Q_{1}A_{1}^{\prime } & Q_{1}^{\prime }B_{1}^{\prime } & -E_{2}R_{2} & -Q_{2}A_{2} & -Q_{2}^{\prime }B_{2} & 0 & 0 & 0 & L_{5}\\ 0 & 0 & 0 & E_{2}R_{3} & Q_{2}A_{2}^{\prime } & Q_{2}^{\prime }B_{2}^{\prime } & -E_{3}R_{3} & -Q_{3}A_{3} & -Q_{3}^{\prime }B_{3} & L_{6}\\ E_{1}^{\prime }R_{2} & 0 & 0 & -E_{2}^{\prime }R_{2} & 0 & 0 & 0 & 0 & 0 & L_{7}\\ 0 & 0 & 0 & E_{2}^{\prime }R_{3} & 0 & 0 & -E_{3}^{\prime }R_{3} & 0 & 0 & L_{8}\\ F_{1} & -F_{1}^{\prime } & F_{1}^{\prime \prime } & F_{2} & -F_{2}^{\prime } & F_{2}^{\prime \prime } & F_{3} & -F_{3}^{\prime } & F_{3}^{\prime \prime } & L_{9}\\ T_{1}(R_{2}-R_{1}) & T_{2}(A_{1}^{\prime }-A_{1}) & T_{3}(B_{1}^{\prime }-B_{1}) & T_{4} & T_{5} & T_{6} & T_{7}(R_{3}-R_{4}) & T_{8}(A_{3}-A_{3}^{\prime }) & T_{9}(B_{3}-B_{3}^{\prime }) & L_{10} \end{array}\right\}$$

(38) $$\begin{array}{ccc} X=\left\{\begin{array}{l} M_{1}\\ M_{2}\\ M_{3}\\ M_{4}\\ M_{5}\\ M_{6}\\ M_{7}\\ M_{8}\\ M_{9}\\ M_{10}+V_{0} \end{array}\right\} & and & Y=\left\{\begin{array}{l} C_{2}^{\left(1\right)}\\ C_{3}^{\left(1\right)}\\ C_{4}^{\left(1\right)}\\ C_{2}^{\left(2\right)}\\ C_{3}^{\left(2\right)}\\ C_{4}^{\left(2\right)}\\ C_{2}^{\left(3\right)}\\ C_{3}^{\left(3\right)}\\ C_{4}^{\left(3\right)}\\ C_{5} \end{array}\right\} \end{array}$$

where $A_{i},A_{i}^{\prime },B_{i},B_{i}^{\prime },Q_{i},Q_{i}^{\prime },E_{i},E_{i}^{\prime },F_{i},F_{i}^{\prime },F_{i}^{\prime \prime },T_{j},L_{k}$ and $M_{k}$ (*i* = 1~3, *j* = 1~9, *k* = 1~10) are given in Appendix A.

Besides, $C_{6k}$ and $D_{k}$ (*k* = 1~3) could also be determined as:

(39a) $$\begin{array}{l} C_{6}^{\left(1\right)}=-\left(2g_{31}^{\left(1\right)}+2g_{33}^{\left(1\right)}\right)R_{1}C_{2}^{\left(1\right)}-\left(g_{31}^{\left(1\right)}-\frac{g_{33}^{\left(1\right)}}{s}\right)(-s+1)R_{1}^{-s}C_{3}^{\left(1\right)}-\left(g_{31}^{\left(1\right)}+\frac{g_{33}^{\left(1\right)}}{s}\right)(s+1)R_{1}^{s}C_{4}^{\left(1\right)}\\ +\left(\frac{{\left(g_{33}^{\left(1\right)}\right)}^{2}}{s_{33}^{D(1)}}+\zeta_{33}^{\left(1\right)}\right)\ln R_{1}C_{5}-H\left[g_{31}^{\left(1\right)}\left(2R_{1}\ln R_{1}+R_{1}\right)+g_{33}^{\left(1\right)}\left(2R_{1}\ln R_{1}-R_{1}\right)\right]-\rho^{\left(1\right)}R_{1}\left(T_{\text{i}}-\frac{C}{\kappa^{\left(1\right)}}\right) \end{array}$$

(39b) $$\begin{array}{l} C_{6}^{\left(2\right)}=\left(2g_{31}^{\left(1\right)}+2g_{33}^{\left(1\right)}\right)\left(R_{2}-R_{1}\right)C_{2}^{\left(1\right)}+\left(g_{31}^{\left(1\right)}-\frac{g_{33}^{\left(1\right)}}{s}\right)\left(A_{1}^{\prime }-A_{1}\right)C_{3}^{\left(1\right)}+\left(g_{31}^{\left(1\right)}+\frac{g_{33}^{\left(1\right)}}{s}\right)\left(B_{1}^{\prime }-B_{1}\right)C_{4}^{\left(1\right)}\\ +\left[\left(\frac{{\left(g_{33}^{\left(1\right)}\right)}^{2}}{s_{33}^{D(1)}}+\zeta_{33}^{\left(1\right)}\right)\ln\frac{R_{1}}{R_{2}}+\left(\frac{{\left(g_{33}^{\left(2\right)}\right)}^{2}}{s_{33}^{D(2)}}+\zeta_{33}^{\left(2\right)}\right)\ln R_{2}-g_{33}^{\left(2\right)}f_{3}\left(R_{2}\right)-f_{4}\left(R_{2}\right)\right]C_{5}\\ -\left(\sum_{i=0}^{N}\frac{2J_{i}}{i+1}R_{2}^{i+1}+2g_{33}^{\left(2\right)}R_{2}\right)C_{2}^{\left(2\right)}+\left(-s+1\right)R_{2}^{-s}\left(\sum_{i=0}^{N}\frac{sJ_{i}}{i-s}R_{2}^{i}+\frac{g_{33}^{\left(2\right)}}{s}\right)C_{3}^{\left(2\right)}\\ -\left(s+1\right)R_{2}^{s}\left(\sum_{i=0}^{N}\frac{sJ_{i}}{i+s}R_{2}^{i}+\frac{g_{33}^{\left(2\right)}}{s}\right)C_{4}^{\left(2\right)}\\ +H\left\{g_{31}^{\left(1\right)}\left[\left(2R_{2}\ln R_{2}+R_{2}\right)-\left(2R_{1}\ln R_{1}+R_{1}\right)\right]+g_{33}^{\left(1\right)}\left[\left(2R_{2}\ln R_{2}-R_{2}\right)-\left(2R_{1}\ln R_{1}-R_{1}\right)\right]\right\}\\ -H\left\{\sum_{i=0}^{N}\left[\frac{3J_{i}}{i+1}R_{2}^{i+1}+\frac{2J_{i}}{i+1}R_{2}^{i+1}\left(\ln R_{2}-\frac{1}{i+1}\right)\right]+g_{33}^{\left(2\right)}\left(2R_{2}\ln R_{2}-R_{2}\right)\right\}\\ +\rho^{\left(1\right)}\left[\left(R_{2}-R_{1}\right)\left(T_{\text{i}}-\frac{C}{\kappa^{\left(1\right)}}\right)+\frac{C}{\kappa^{\left(1\right)}}R_{2}\ln\frac{R_{2}}{R_{1}}\right]-\rho^{\left(2\right)}R_{2}\left[T_{\text{i}}+\frac{C}{\kappa^{\left(1\right)}}\ln\frac{R_{2}}{R_{1}}-\frac{C}{\kappa^{\left(2\right)}}\right] \end{array}$$

(39c) $$\begin{array}{l} C_{6}^{\left(3\right)}=V_{0}-\left(2g_{31}^{\left(3\right)}+2g_{33}^{\left(3\right)}\right)R_{4}C_{2}^{\left(3\right)}-\left(g_{31}^{\left(3\right)}-\frac{g_{33}^{\left(3\right)}}{s}\right)(-s+1)R_{4}^{-s}C_{3}^{\left(3\right)}-\left(g_{31}^{\left(3\right)}+\frac{g_{33}^{\left(3\right)}}{s}\right)(s+1)R_{4}^{s}C_{4}^{\left(3\right)}\\ +\left(\frac{{\left(g_{33}^{\left(3\right)}\right)}^{2}}{s_{33}^{D(3)}}+\zeta_{33}^{\left(3\right)}\right)\ln R_{4}C_{5}-H\left[g_{31}^{\left(3\right)}\left(2R_{4}\ln R_{4}+R_{4}\right)+g_{33}^{\left(3\right)}\left(2R_{4}\ln R_{4}-R_{4}\right)\right]\\ -\rho^{\left(3\right)}R_{4}\left(T_{\text{i}}+\frac{C}{\kappa^{\left(1\right)}}\ln\frac{R_{2}}{R_{1}}+\frac{C}{\kappa^{\left(2\right)}}\ln\frac{R_{3}}{R_{2}}+\frac{C}{\kappa^{\left(3\right)}}\ln\frac{R_{4}}{R_{3}}-\frac{C}{\kappa^{\left(3\right)}}\right) \end{array}$$

(40a) $$D_{1}=0$$

(40b) $$\begin{array}{l} D_{2}=-\left(2s_{13}^{D(2)}+2s_{33}^{D(2)}\right)R_{0}C_{2}^{\left(2\right)}-\left(s_{13}^{D(2)}-\frac{s_{33}^{D(2)}}{s}\right)\left(-s+1\right)R_{0}^{-s}C_{3}^{\left(2\right)}-\left(s_{13}^{D(2)}+\frac{s_{33}^{D(2)}}{s}\right)\left(s+1\right)R_{0}^{s}C_{4}^{\left(2\right)}\\ -\left[s_{13}^{D(2)}f_{2}^{\prime }\left(R_{0}\right)+s_{33}^{D(2)}f_{3}\left(R_{0}\right)-\frac{s_{13}^{D(2)}g_{33}^{\left(2\right)}}{s_{33}^{D(2)}}+J_{0}\right]C_{5}\\ -H\left[s_{13}^{D(2)}\left(2R_{0}\ln R_{0}+R_{0}\right)+s_{33}^{D(2)}\left(2R_{0}\ln R_{0}-R_{0}\right)\right]\\ -\alpha_{r}^{\left(2\right)}R_{0}\left(T_{\text{i}}+\frac{C}{\kappa^{\left(1\right)}}\ln\frac{R_{2}}{R_{1}}+\frac{C}{\kappa^{\left(2\right)}}\ln\frac{R_{0}}{R_{2}}-\frac{C}{\kappa^{\left(2\right)}}\right) \end{array}$$

(40c) $$\begin{array}{l} D_{\text{3}}=-\frac{\pi }{\text{2}}\left[\left(2s_{1\text{1}}^{D(2)}-\text{2}s_{33}^{D(2)}\right)C_{2}^{\left(2\right)}\text{+}J_{1}C_{5}+s_{11}^{D(2)}H\left(2\ln R_{0}+3\right)-s_{33}^{D(2)}H\left(2\ln R_{0}-1\right)\right]\\ -\left(\alpha_{\theta }^{\left(2\right)}-\alpha_{r}^{\left(2\right)}\right)\frac{\pi }{2}\left(T_{\text{i}}+\frac{C}{\kappa^{\left(1\right)}}\ln\frac{R_{2}}{R_{1}}+\frac{C}{\kappa^{\left(2\right)}}\ln\frac{R_{0}}{R_{2}}\right)-\alpha_{r}^{\left(2\right)}\frac{\pi }{2}\frac{C}{\kappa^{\left(2\right)}} \end{array}$$

In summary, the electroelastic fields in each individual layer of the curved FGP actuator with the consideration of thermal effect are determined. 

## 4. Results and Discussion

As mentioned before, all the material properties except the piezoelectric coefficient *g*_31_ are assumed as constants in the current work. The elastic, piezoelectric (except *g*_31_), dielectric constants, thermal expansion and pyroelectric coefficients for the different layers of the sandwiched piezoelectric actuator are taken as those for the *PZT*-4 [28,31] in the numerical calculation, and the typical value of thermal conductivity is taken as $\kappa =2.1{\text{Wm}}^{-\text{1}}{\text{K}}^{-\text{1}}$. All these material constants are listed in Table 1. It should be mentioned that the derived solutions in the previous section are applicable for any arbitrary format of g31 with Taylor series expansion. For case study of the actuator configuration in Figure 1, the middle FGM layer is assumed as exponentially graded along the radial direction as shown in Equation (10), and the coefficients of Taylor expansion of g31 can be determined from its values at the boundaries of the FGM layer. The upper layer of the sandwich structure is taken as PZT-4 and its piezoelectric constant is $g_{31}(R_{3})=g_{31}^{\left(3\right)}=-17.8\times 10^{-3}{\text{m}}^{2}{\text{C}}^{-1}$, while the piezoelectric constant for the lower layer is assumed as $g_{31}(R_{2})=g_{31}^{\left(1\right)}=-9.35\times 10^{-3}{\text{m}}^{2}{\text{C}}^{-1}$. The geometry of the curved FGP actuator with sandwich structure is fixed with *R*_1_ = 15.5 mm, *R*_2_ = 16.0 mm,* R*_3_ = 17.0 mm and *R*_4_ = 17.5 mm.

**Table 1:** Material Constants of PZT-4 (*g*-type constitutive relations).

Elastic constant (10^−12^m^−2^N^−1^)				Piezoelectric constant (10^−3^m^2^C^−1^)		Dielectric constant (10^6^mF^−1^)		Thermal expansion (10^−6^K^−1^)		Pyroelectric constant (10^3^NC^−1^K^−1^)	Thermal conductivity (Wm^−1^K^−1^)
$s_{11}^{D}$	$s_{13}^{D}$	$s_{33}^{D}$	$s_{44}^{D}$	$g_{33}$	$g_{15}$	$\zeta_{11}$	$\zeta_{33}$	$\alpha_{\theta }$	$\alpha_{r}$	ρ	κ
7.95	−3.03	7.91	17.91	23.91	40.36	76.87	99.65	2.89	1.29	5.56	2.1

**Figure 2:**
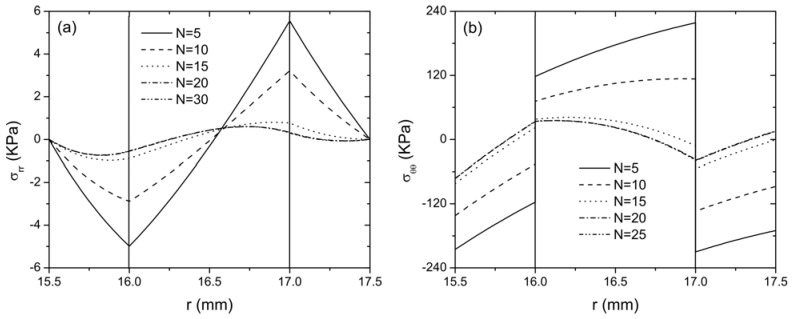
The distribution of stresses along radial direction for the curved FGP actuator when *V*_0_ = 100 V and *T*_o_ = 10 °C (**a**) radial stress; (**b**) hoop stress.

**Figure 3:**
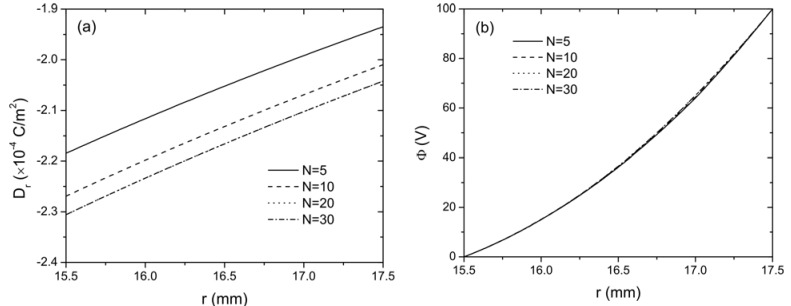
The distribution of (**a**) electric displacement *D_r_* and (**b**) electric potential *Φ* along the radial direction of the curved FGP actuator when *V*_0_ = 100 V.

For different values of *N* (5, 10, 15, 20 and 30 for example) in the Taylor series expansion of the exponential format *g*_31_, the distribution of radial stress $\sigma_{rr}$ and hoop stress $\sigma_{\theta \theta }$ along the radial direction of the actuator is plotted in Figure 2 when the actuator is subjected to an electric potential *V*_0_ = 100 V and the temperature rise at the upper surface of layer 3 and the lower surface of layer 1 is* T*_o_ = 10 °C and *T*_i_ = 0 °C, respectively. From these two figures, it is clearly illustrated that the curves are almost identical for *N* = 20 and 30. Therefore, it can be concluded that convergence is obtained when using the Taylor series up to 20 terms to expand the piezoelectric coefficient *g*_31_(*r*) in the current case study. Taking different values of *N* is equivalent to the change of material gradient for the FGP layer, therefore, the discrepancy among the curves with different *N* indicates the stress distribution is significantly affected by the material gradient. It is seen from Figure 2(b) that the hoop stress will be continuous at the interfaces when *N* = 20 and 30, unlike an abrupt change for a smaller *N*, indicating the discontinuity of hoop stresses at the interfaces of dissimilar media can be eliminated by using graded material, which may prevent the possible failure of the structures at the interfaces. In addition, non-zero stresses $\left(\sigma_{rr},\sigma_{\theta \theta }\right)$ are always observed in this sandwich actuator with thermal conduction. These internal stresses should be considered in the design of curved FGP actuator with sandwich structure. The distribution of electric displacement *D_r_* and electric potential *Φ* along the radial direction are shown in Figure 3 with different *N*. It is observed that the material gradient has relatively large influence on the electric displacement but no significant influence on electric potential distribution as *Φ* is almost identical for different values of *N*.

**Figure 4:**
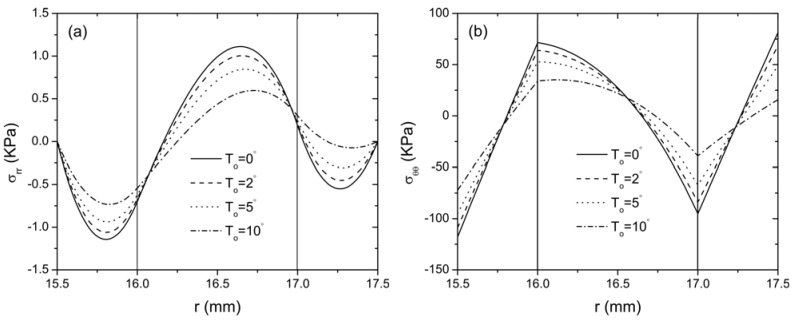
The distribution of stresses along the radial direction for the curved FGP actuator with different *T*_o_ when *V*_0_ = 100 V (**a**) radial stress; (**b**) hoop stress.

**Figure 5:**
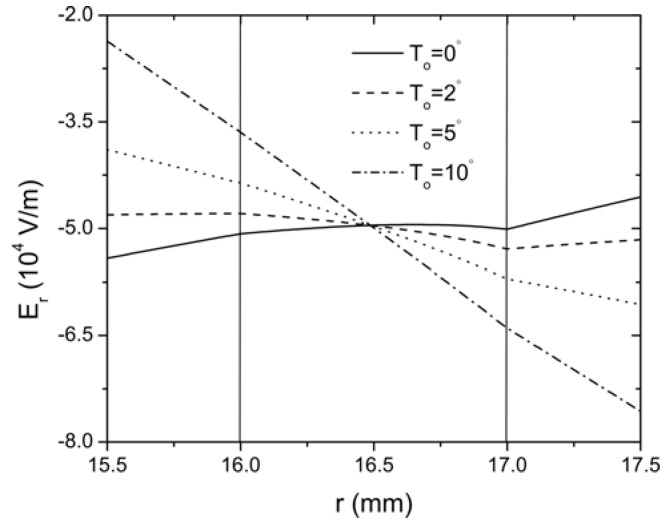
The distribution of the electric field along the radial direction of the curved FGP actuator when *V*_0_=100 V.

**Figure 6:**
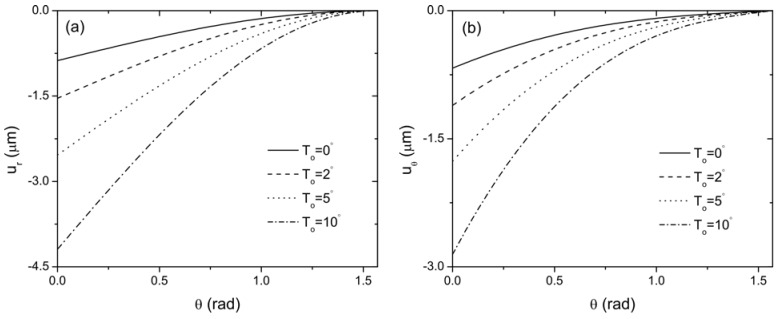
The variation of displacements with the angle *θ* in the middle of the actuator when *V*_0_ = 100 V (**a**) radial displacement; (**b**) hoop displacement.

In the following, *N* is taken as 20 to represent a convergent Taylor series expansion for the exponentially graded FGP layer. Therefore, the middle layer of the sandwich structure is continuously jointed with the lower and the upper layer. The effect of thermal loading on the distribution of radial and hoop stresses of this curved sandwich actuator are plotted in Figure 4 for different temperatures *T*_o_ = (0 °C, 2 °C, 5 °C, 10 °C), while the temperature on the inner surface of layer 1 is kept constant. It is clearly indicated that the thermal loading has a significant effect on the stress distribution in the curved actuator. With the increase of the temperature, the magnitude of the stresses decreases. Under the same loading condition, the distribution of the electric field in the actuator is presented in Figure 5. It is seen that the thermal conduction significantly changes the distribution of electric field as expected, *i.e.*, the electric field increases with the increase of the thermal loading. The influence of thermal loading can also be observed from the distribution of radial displacement $u_{r}$ and hoop displacement $u_{\theta }$ along the circumferential direction of the curved actuator as shown in Figure 6. The significant effect of thermal loading on the electroelastic fields of the curved FGP actuator observed from these figures indicate that it is necessary to consider the thermal effect in the design and optimization of the curved FGP sandwich actuator.

The current FGP actuator with sandwich structure can be easily reduced to a conventional piezoelectric bimorph by setting the lower layers 1 and 2 as the same material different from the upper layer 3 with $g_{31}=-9.35\times 10^{-3}{\text{m}}^{2}{\text{C}}^{-1}$ , while the upper layer 3 is assumed to have the piezoelectric coefficient as $g_{31}=-17.8\times 10^{-3}{\text{m}}^{2}{\text{C}}^{-1}$. It will be interesting to compare the stresses and displacements of the current FGP actuator with sandwich structure to those of a conventional piezoelectric bimorph actuator under the same thermal and electrical loadings. 

**Figure 7:**
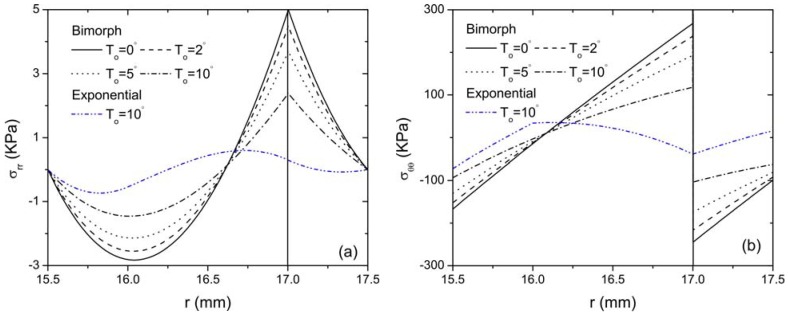
Comparison of the distribution of stresses along the radial direction for bimorph and FGP sandwich actuator when *V*_0_ = 100 V (**a**) radial stress; (**b**) hoop stress.

**Figure 8:**
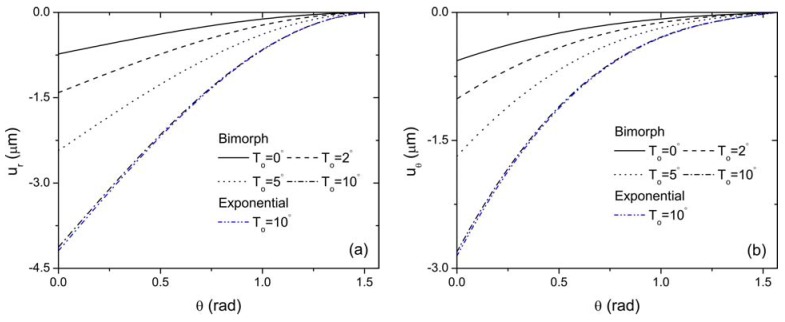
Comparison of the variation of displacements with *θ* for bimorph and FGP sandwich actuator when *V*_0_ = 100 V (**a**) radial displacement; (**b**) hoop displacement.

Figure 7 shows the distribution of radial and hoop stresses of the bimorph actuator along the radial direction for different thermal loading conditions. It is observed that the thermal effect on the stress field of the bimorph is also prominent. A sudden change of variation trend of the radial stress with *r* occurs at the interface of the bimorph, while the hoop stress is discontinuous at the same interface. The variation of the radial and hoop stress of the FGP actuator with sandwich structure is also provided for comparison when *T*_o_ = 10 °C. It is clearly indicated that the magnitude of both stresses reduces drastically compared with those of the bimorph actuator. The curves obtained are smoothly changing along the radial direction of the actuator and no sharp peaks are observed at the interface for the FGP actuator. The distribution of the displacement fields in the middle of both piezoelectric bimorph and FGP sandwich actuator is depicted in Figure 8 for comparison. It is observed that the FGP sandwich actuator provides relative larger displacements compared to piezoelectric bimorph actuator for the same loading conditions. Tabular results are also provided to supplement the graphic presentation for stress and displacement distribution as shown in Table 2. For example, the radial stress at r = 17 mm (*i.e.*, the interface of the bimorph) for both the bimorph and FGP actuators under different thermal loads are quantitatively shown in this table. It is seen that, by using the FGP sandwich actuator, the magnitude of the radial stress in the bimorph actuator has decreased significantly as illustrated by percentage. Also larger displacement at the free end of the FGP sandwich actuator is always observed compared to that of the bimorph actuator under different thermal loads. Moreover, with the decrease of the temperature rise T_o_, the difference of the free end displacements of these two type actuators increases. Based on these graphical displays and tabular data, the advantages of using FGP sandwich actuator over bimorph actuator are clearly demonstrated. Therefore, it is concluded that FGPMs are very important for the design and optimization of an actuator by generating less internal stresses while providing larger deflections for actuation. In addition, the thermal conduction has a significant effect on the electroelastic fields of the piezoelectric structure, which should also be considered for the design purpose of curved FGP actuator. 

**Table 2:** Comparison of radial stress and free end displacements of the bimorph and FGP sandwich actuators under different thermal loads when *V*_0_ = 100 V.

Thermal loads *T*_o_ (°C)	${\sigma_{rr}|}_{r=17\text{ mm}}$ (KPa)		Difference (%)	${u_{r}|}_{\theta =0^{\circ }}$ (μm)		Difference (%)	${u_{\theta }|}_{\theta =0^{\circ }}$ (μm)		Difference (%)
	Bimorph	FGP		Bimorph	FGP		Bimorph	FGP	
0	4.983	0.184	−96.307	−0.731	−0.877	+19.973	−0.565	−0.673	+19.115
2	4.462	0.207	−95.361	−1.410	−1.540	+9.220	−1.014	−1.110	+9.467
5	3.680	0.243	−93.397	−2.429	−2.534	+4.323	−1.687	−1.764	+4.564
10	2.377	0.302	−87.295	−4.128	−4.191	+1.526	−2.808	−2.855	+1.674

## 5. Conclusions

In this work, a theoretical analysis of a curved functionally graded piezoelectric actuator with sandwich structure under electrical and thermal loads is conducted. The piezoelectric coefficient *g*_31_ of the FGP layer is assumed to vary exponentially along the radial direction. By using Airy stress function, the electroelastic fields of the actuator are obtained analytically. Simulation results are presented to show the influence of material gradient and thermal conduction on the curved actuator configuration. It is found that the material gradient has a significant influence on the stresses and electric displacement, but not on the electric potential. However, thermal conduction has a significant effect on all the electroelastic fields of the curved FGP actuator with sandwich structure. By comparing the FGP sandwich actuator with piezoelectric bimorph, it is clearly indicated that much smaller internal stresses with no compromise of deflections could be achieved by the FGP sandwich actuator. This work is expected to provide helpful guidelines for the design and optimization of curved piezoelectric actuator with the consideration of thermal effect.

## Appendix A

The parameters in the matrix of Equation (38) are given below:

(A1) $$A_{k}=(-s+1)R_{k}^{-s};A_{k}^{\prime }=(-s+1)R_{k+1}^{-s}$$

(A2) $$B_{k}=(s+1)R_{k}^{s};B_{k}^{\prime }=(s+1)R_{k+1}^{s}$$

(A3) $$Q_{k}=s_{13}^{D(k)}-s_{33}^{D(k)}/s;Q_{k}^{\prime }=s_{13}^{D(k)}+s_{33}^{D(k)}/s$$

(A4) $$E_{k}=2s_{13}^{D(k)}+2s_{33}^{D(k)};E_{k}^{\prime }=2s_{11}^{D(k)}-2s_{33}^{D(k)}$$

(A5) $$F_{k}=R_{k+1}^{2}-R_{k}^{2};F_{k}^{\prime }=s(R_{k+1}^{-s+1}-R_{k}^{-s+1});F_{k}^{\prime \prime }=s(R_{k+1}^{s+1}-R_{k}^{s+1})$$

(A6) $$\begin{array}{l} T_{1}=2g_{31}^{\left(1\right)}+2g_{33}^{\left(1\right)},T_{2}=g_{31}^{\left(1\right)}-g_{33}^{\left(1\right)}/s,T_{3}=g_{31}^{\left(1\right)}+g_{33}^{\left(1\right)}/s;\\ T_{4}=\sum_{i=0}^{N}\frac{2J_{i}}{i+1}\left(R_{3}^{i+1}-R_{2}^{i+1}\right)+2g_{33}^{\left(2\right)}\left(R_{3}-R_{2}\right);\\ T_{5}=(-s+1)\left[\sum_{i=0}^{N}\frac{sJ_{i}}{i-s}\left(R_{2}^{-s+i}-R_{3}^{-s+i}\right)+\frac{g_{33}^{\left(2\right)}}{s}\left(R_{2}^{-s}-R_{3}^{-s}\right)\right];\\ T_{6}=(s+1)\left[\sum_{i=0}^{N}\frac{sJ_{i}}{i+s}\left(R_{3}^{s+i}-R_{2}^{s+i}\right)+\frac{g_{33}^{\left(2\right)}}{s}\left(R_{3}^{s}-R_{2}^{s}\right)\right];\\ T_{7}=-2g_{31}^{\left(3\right)}-2g_{33}^{\left(3\right)},T_{8}=-g_{31}^{\left(3\right)}+g_{33}^{\left(3\right)}/s,T_{9}=-g_{31}^{\left(3\right)}-g_{33}^{\left(3\right)}/s \end{array}$$

(A7) L1=−g33(1)/s33D(1);L2=−g33(3)/s33D(3);L3=−f2′(R2);L4=f2′(R3);L5=g31(1)−s13D(2)f2′(R2)−s33D(2)f3(R2)−J0;L6=−g31(3)+s13D(2)f2′(R3)+s33D(2)f3(R3)+J0;L7=−J1R2;L8=J1R3;L9=1s11D(2)∑i=2NJii2s2−i2R3i+1−R2i+1i+1;L10=((g33(3))2s33D(3)+ζ33(3))lnR1R4−g33(2)[f3(R2)−f3(R3)]−[f4(R2)−f4(R3)]

(A8) $$\begin{array}{l} M_{1}=-H\left(2R_{1}\ln R_{1}+R_{1}\right);M_{2}=-H\left(2R_{4}\ln R_{4}+R_{4}\right);M_{3}=M_{4}=0;\\ M_{5}=H\left[\left(s_{13}^{D(2)}-s_{13}^{D(1)}\right)\left(2R_{2}\ln R_{2}+R_{2}\right)+\left(s_{33}^{D(2)}-s_{33}^{D(1)}\right)\left(2R_{2}\ln R_{2}-R_{2}\right)\right]\\ +\left(\alpha_{r}^{\left(2\right)}-\alpha_{r}^{\left(1\right)}\right)\left(T_{\text{i}}+\frac{C}{\kappa^{\left(1\right)}}\ln\frac{R_{2}}{R_{1}}\right)R_{2}+CR_{2}\left(\frac{\alpha_{r}^{\left(1\right)}}{\kappa^{\left(1\right)}}-\frac{\alpha_{r}^{\left(2\right)}}{\kappa^{\left(2\right)}}\right);\\ M_{6}=H\left[\left(s_{13}^{D(3)}-s_{13}^{D(2)}\right)\left(2R_{3}\ln R_{3}+R_{3}\right)+\left(s_{33}^{D(3)}-s_{33}^{D(2)}\right)\left(2R_{3}\ln R_{3}-R_{3}\right)\right]\\ +\left(\alpha_{r}^{\left(3\right)}-\alpha_{r}^{\left(2\right)}\right)\left(T_{\text{i}}+\frac{C}{\kappa^{\left(1\right)}}\ln\frac{R_{2}}{R_{1}}+\frac{C}{\kappa^{\left(2\right)}}\ln\frac{R_{3}}{R_{2}}\right)R_{3}+CR_{3}\left(\frac{\alpha_{r}^{\left(2\right)}}{\kappa^{\left(2\right)}}-\frac{\alpha_{r}^{\left(3\right)}}{\kappa^{\left(3\right)}}\right);\\ M_{7}=H\left[\left(s_{11}^{D(2)}-s_{11}^{D(1)}\right)\left(2R_{2}\ln R_{2}+3R_{2}\right)-\left(s_{33}^{D(2)}-s_{33}^{D(1)}\right)\left(2R_{2}\ln R_{2}-R_{2}\right)\right]\\ +\left[\alpha_{\theta }^{\left(2\right)}-\alpha_{r}^{\left(2\right)}-\left(\alpha_{\theta }^{\left(1\right)}-\alpha_{r}^{\left(1\right)}\right)\right]\left(T_{\text{i}}+\frac{C}{\kappa^{\left(1\right)}}\ln\frac{R_{2}}{R_{1}}\right)R_{2}+CR_{2}\left(\frac{\alpha_{r}^{\left(2\right)}}{\kappa^{\left(2\right)}}-\frac{\alpha_{r}^{\left(1\right)}}{\kappa^{\left(1\right)}}\right);\\ M_{8}=H\left[\left(s_{11}^{D(3)}-s_{11}^{D(2)}\right)\left(2R_{3}\ln R_{3}+3R_{3}\right)-\left(s_{33}^{D(3)}-s_{33}^{D(2)}\right)\left(2R_{3}\ln R_{3}-R_{3}\right)\right]\\ +\left[\alpha_{\theta }^{\left(3\right)}-\alpha_{r}^{\left(3\right)}-\left(\alpha_{\theta }^{\left(2\right)}-\alpha_{r}^{\left(2\right)}\right)\right]\left(T_{\text{i}}+\frac{C}{\kappa^{\left(1\right)}}\ln\frac{R_{2}}{R_{1}}+\frac{C}{\kappa^{\left(2\right)}}\ln\frac{R_{3}}{R_{2}}\right)R_{3}+CR_{3}\left(\frac{\alpha_{r}^{\left(3\right)}}{\kappa^{\left(3\right)}}-\frac{\alpha_{r}^{\left(2\right)}}{\kappa^{\left(2\right)}}\right);\\ M_{9}=H\left[R_{1}^{2}\left(\ln R_{1}+1\right)-R_{4}^{2}\left(\ln R_{4}+1\right)\right];\\ M_{10}=H\left\{g_{31}^{\left(1\right)}\left[\left(2R_{1}\ln R_{1}+R_{1}\right)-\left(2R_{2}\ln R_{2}+R_{2}\right)\right]+g_{33}^{\left(1\right)}\left[\left(2R_{1}\ln R_{1}-R_{1}\right)-\left(2R_{2}\ln R_{2}-R_{2}\right)\right]\right\}\\ +H\left\{g_{33}^{\left(2\right)}\left[\left(2R_{2}\ln R_{2}-R_{2}\right)-\left(2R_{3}\ln R_{3}-R_{3}\right)\right]\right\}\\ +H\left\{\sum_{i=0}^{N}\frac{3J_{i}}{i+1}\left(R_{2}^{i+1}-R_{3}^{i+1}\right)+\frac{2J_{i}}{i+1}\left[R_{2}^{i+1}\left(\ln R_{2}-\frac{1}{i+1}\right)-R_{3}^{i+1}\left(\ln R_{3}-\frac{1}{i+1}\right)\right]\right\}\\ +H\left\{g_{31}^{\left(3\right)}\left[\left(2R_{3}\ln R_{3}+R_{3}\right)-\left(2R_{4}\ln R_{4}+R_{4}\right)\right]+g_{33}^{\left(3\right)}\left[\left(2R_{3}\ln R_{3}-R_{3}\right)-\left(2R_{4}\ln R_{4}-R_{4}\right)\right]\right\}\\ +\rho^{\left(1\right)}\left[\left(R_{1}-R_{2}\right)\left(T_{\text{i}}-\frac{C}{\kappa^{\left(1\right)}}\right)+\frac{C}{\kappa^{\left(1\right)}}R_{2}\ln\frac{R_{1}}{R_{2}}\right]\\ +\rho^{\left(2\right)}\left[\left(R_{2}-R_{3}\right)\left(T_{\text{i}}+\frac{C}{\kappa^{\left(1\right)}}\ln\frac{R_{2}}{R_{1}}-\frac{C}{\kappa^{\left(2\right)}}\right)+\frac{C}{\kappa^{\left(2\right)}}R_{3}\ln\frac{R_{2}}{R_{3}}\right]\\ +\rho^{\left(3\right)}\left[\left(R_{3}-R_{4}\right)\left(T_{\text{i}}+\frac{C}{\kappa^{\left(1\right)}}\ln\frac{R_{2}}{R_{1}}+\frac{C}{\kappa^{\left(2\right)}}\ln\frac{R_{3}}{R_{2}}-\frac{C}{\kappa^{\left(3\right)}}\right)+\frac{C}{\kappa^{\left(3\right)}}R_{4}\ln\frac{R_{3}}{R_{4}}\right] \end{array}$$
